# Successful Repair of a Ruptured Common Iliac Aneurysm with Associated Arteriovenous Fistula Using Aneurysm Wall

**DOI:** 10.3400/avd.cr.23-00122

**Published:** 2024-03-13

**Authors:** Yusuke Nakata, Kazuyuki Miyamoto

**Affiliations:** 1 Department of Cardiovascular Surgery, Fukuoka Red Cross Hospital, Fukuoka, Fukuoka, Japan

**Keywords:** common iliac artery, arteriovenous fistula, ruptured aneurysm

## Abstract

Ruptured abdominal aortic aneurysms and common iliac artery aneurysms (CIAAs) are rarely associated with an arteriovenous fistula (AVF). In such cases, surgery is frequently extremely difficult and the prognosis is usually poor. We report a case of a ruptured CIAA with a common iliac AVF in a 58-year-old male patient who presented with symptoms of severe edema in his left lower extremity. We used an aneurysm wall patch to repair the fistula and successfully reconstruct the common iliac vein, and a bifurcated prosthetic graft for abdominal aortic and iliac artery replacement.

## Introduction

A ruptured common iliac artery aneurysm (CIAA) rarely develops into an arteriovenous fistula (AVF). Most patients have symptoms associated with high-output heart failure, often presenting with symptoms such as lower leg edema and lymphorrhea. If a preoperative diagnosis cannot be made, surgery is usually difficult and the prognosis is extremely poor.^1)^ Herein, we report the successful repair of a ruptured CIAA with a large AVF using an aneurysm wall patch.

## Case Report

**Figure 1:**
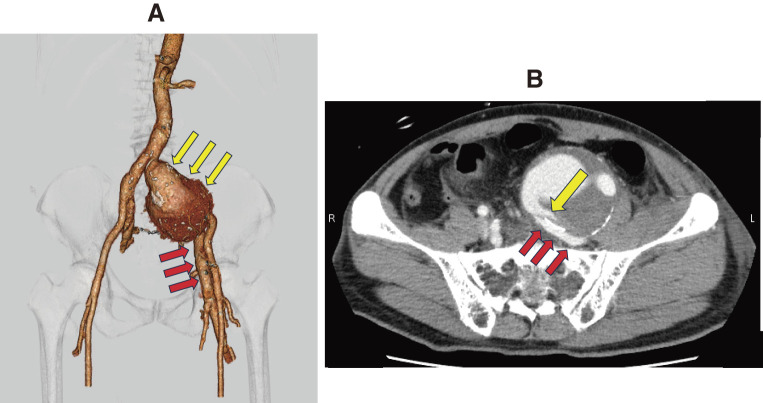
Fig. 1 (**A** and **B**) Preoperative computed tomography. Three-dimensional computed tomography reveals a large CIAA (yellow arrows). The distal end of the left CIV (red arrows) markedly enhances in the arterial phase, but the proximal end does not (**A**). Coronal CT reveals a large shunt between the common iliac artery and vein (yellow arrow). Red arrows demonstrate that the left CIAA compresses the left CIV (**B**). CIAA: common iliac artery aneurysm; CIV: common iliac vein; CT: contrast-enhanced computed tomography

A 58-year-old male was referred to our institution from a nearby hospital owing to symptoms of severe edema in his left lower extremity, which suggested an impending rupture of a large CIAA. At the time of admission, the left lower leg edema had worsened and urgent surgery was scheduled. His hemodynamics were stable, with a blood pressure of 129/60 mmHg and a heart rate of 92 beats per minute. Chest radiography showed no pulmonary congestion, and echocardiography showed a left ventricular end-diastolic diameter/end-systolic diameter of 51/32 mm, a left ventricular ejection fraction of 67%, no significant valvular disease, and no evidence of heart failure. A pulsating abdominal mass was palpable in the left lower abdomen and a continuous bruit was heard. Redness, swelling, and pain were observed in the left lower extremity, from the thigh to the toes. A lower extremity duplex scan revealed no mobile deep vein thrombus, but arterial blood flow was observed in the common femoral vein. Contrast-enhanced computed tomography (CT) revealed a left CIAA (72 mm in diameter). Additionally, contrast enhancement of the left common iliac vein (CIV) was observed in the early phase, suggesting an AVF between the CIA and CIV. Owing to the pressure caused by the aneurysm, almost no contrast enhancement was seen in the inferior vena cava (**Fig. 1**).

**Figure 2:**
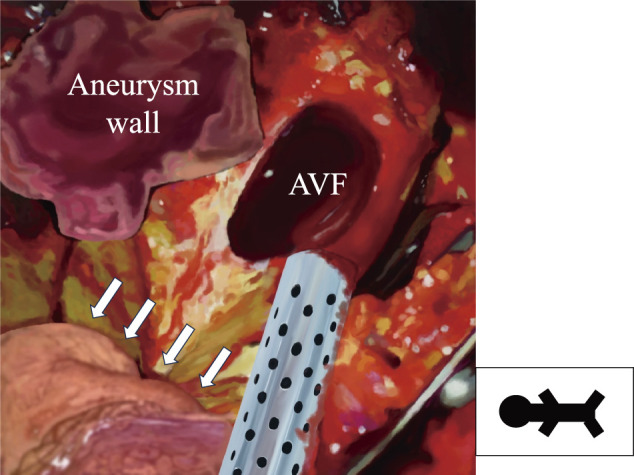
Fig. 2 Intraoperative schema. In this view, the left side is cephalad and the right side is caudad. White arrows show a part of the sigmoid colon. A cell saver as an autologous blood collection device is used to control bleeding from the AVF, and patch closure of the AVF is performed using the aneurysm wall. AVF: arteriovenous fistula

Urgent surgery was performed via a midline abdominal incision. A retroperitoneal hematoma was observed in the left lower abdomen. Although there was almost no adhesion, the left ureter was compressed by the left CIAA. A cross-clamp was applied to the infrarenal aorta and right CIA. The left internal and external iliac arteries could not be identified because of the presence of the large aneurysm. An additional incision was made in the left groin, and a distal clamp was applied to the common femoral artery (CFA). When the CIAA was opened, a large AVF measuring 40 × 30 mm on cross-section was identified between the left common iliac artery and vein. Furthermore, we found that a fresh thrombus measuring 10 × 40 mm had emerged from within the AVF. Because of the large AVF, it was difficult to control the bleeding; hence, we performed the suture-ligation of the orifice of the left internal iliac artery (IIA) to control the blood from the IIA. The full thickness of the aneurysm wall was then trimmed into a rectangle measuring 50 × 40 mm, and the AVF was closed with a patch using 4-0 polypropylene sutures (**Fig. 2**). During patch closure, we used a cell-saver and manually compressed the proximal and distal ends of the AVF with gauze to ensure a clear view of the surgical field and maintain his hemodynamic status. After patch closure, abdominal aortic replacement was performed using a bifurcated prosthetic graft (J-Graft, 16 × 11 mm; Japan Lifeline, Tokyo, Japan). The proximal end was anastomosed to the infrarenal aorta, the right leg to the right CIA, and the left leg to the left CFA through the left inguinal ligament. The stump of the left external iliac artery was closed using a pledgeted suture. The surgery was completed by placing a drain in the retroperitoneum. The duration of the operation was 6 h and 31 min, the time required for patch closure of the AVF was 23 min, total blood loss was 983 ml, total cell saver blood salvage was 10551 ml, and total blood transfusions administered were 16 units of red blood cells, 16 units of fresh frozen plasma, and 30 units of platelets.

**Figure 3:**
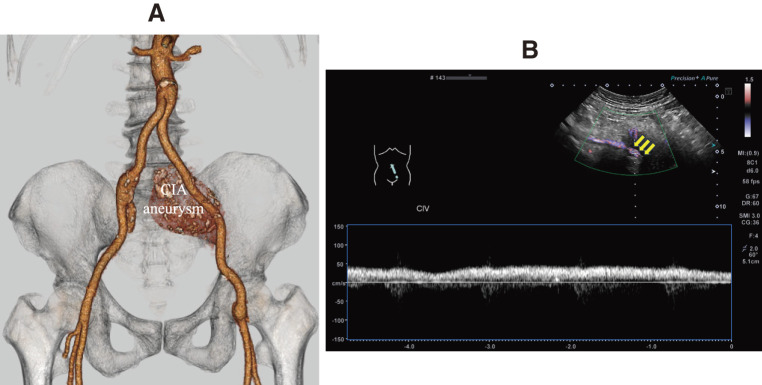
Fig. 3 (**A** and **B**) Postoperative computed tomography and abdominal ultrasonography. Three-dimensional computed tomography reveals good revascularization using a bifurcated prosthetic graft, with traces of the left CIAA remaining (**A**). Abdominal ultrasonography confirms blood flow into the left CIV, which was reconstructed using the aneurysm wall. No significant changes in intravenous flow velocity are observed (yellow arrows) (**B**). CIAA: common iliac artery aneurysm; CIV: common iliac vein

The patient was weaned from the ventilator on the day of the surgery and was able to walk on postoperative day 3. The edema of the left lower extremity decreased postoperatively, along with drainage of the lymphatic fluid from the drain in the left inguinal region. Postoperative contrast-enhanced CT showed good revascularization via the vascular graft (**Fig. 3A**) and no pulmonary artery thromboembolism, while abdominal ultrasonography revealed blood flow through the vein that was patched with the aneurysm wall (**Fig. 3B**). To prevent deep vein thrombosis, direct oral anticoagulant was administered and elastic stockings were applied until he was able to get out of bed. The patient was discharged on postoperative day 15. A follow-up examination one month later showed no problems.

## Discussion

Ruptured abdominal aortic aneurysms and CIAAs rarely develop into AVFs, occurring in only 6% of ruptured aortic aneurysms.^2)^ Ruptured CIAAs with associated AVFs often cannot be detected promptly; delayed diagnosis of a ruptured CIAA occurs in 60% of the cases,^3)^ resulting in poor prognosis. Symptoms vary depending on the shape and size of the aneurysm and include palpable pulsatile masses, as well as high-output heart failure, deep venous thrombosis, lymphorrhea, and venous hypertension, which are common symptoms of an AVF in ruptured aneurysms.^4)^ In our patient, although the CIAA was large, there were a few subjective symptoms until the aneurysm ruptured. Although the AVF had formed due to the rupture of the CIAA, preoperative and postoperative echocardiography showed no change in the cardiac load. The shunt venous pressure was localized in the left lower leg due to venous displacement caused by the large aneurysm, and the patient did not develop heart failure before surgery.

Large amounts of bleeding are expected when an aneurysm with an AVF is incised. For small AVFs, methods such as using a Fogarty occlusion catheter, compressing the iliac veins, and using balloon-tipped catheters to control bleeding from AVFs have been reported^3^^,^^5^^,^^6)^; however, for large AVFs, it is difficult to control bleeding. Extracting the patient’s blood and returning it to the patient via autologous blood transfusion may be necessary. Regarding the surgical method of closing the AVF, there are many case reports in which patch closure was performed using a bovine pericardial patch^5)^ or autologous tissue such as femoral vein^7)^; this is similar to the approach done for our patient, wherein a part of the large aneurysm wall was easily and quickly harvested. The AVF was so large that the aneurysm wall was suitable for use as a patch. There have also been reports of venous ligation being performed owing to the difficulty in stopping the bleeding.^8)^ Because the AVF is located within the aneurysm, there is a risk that the surrounding mural thrombus may flow into the vein when closing the AVF. Therefore, careful thrombus removal from the aneurysm is necessary. When incising an aneurysm, it may be necessary to allow some bleeding to perform sufficient aspiration for autologous blood transfusion to remove blood clots within the AVF and prevent pulmonary thromboembolism. In this patient, it was not necessary to place an inferior vena cava filter or insert a Fogarty balloon catheter into the inferior vena cava before surgery because a thrombus within the AVF could not be confirmed on preoperative examination. However, preparation for occlusion with a balloon in case of bleeding should be considered.

There are also reports of two-stage treatments, such as closure of AVFs using stent grafting therapy.^8^^,^^9)^ However, there are many potential complications to this kind of treatment, such as endoleaks, residual shunts, aneurysm enlargement, and aneurysm rupture. Therefore, these cannot be considered as established treatments. Radical surgical treatment of ruptured abdominal aneurysms associated with AVFs is challenging but inevitable. Urgent surgery is required for ruptured CIAA; however, adequate preoperative examination and treatment strategies are equally significant.

## Conclusion

We performed surgery for a ruptured CIAA with an AVF that was diagnosed due to the unique presentation of unilateral lower leg edema without heart failure. Because of the large AVF, it was extremely difficult to control the bleeding; however, it was repaired quickly using the aneurysm wall. Repair using immediately available aneurysm wall may be useful.

## Informed Consent

The patient provided informed consent for publication of the case report and accompanying images.

## Disclosure Statement

The authors declare no conflict of interest.

## Author Contributions

Study conception: YN

Data collection: YN

Analysis: YN

Investigation: YN

Manuscript preparation: YN

Funding acquisition: YN

Critical review and revision: all authors

Final approval of the article: all authors

Accountability for all aspects of the work: all authors.
